# Discussions of the Mississippi Valley Dental Society, March 5th, 1873

**Published:** 1873-05

**Authors:** 


### Proceedings of Societies.

### DISCUSSIONS OF THE MISSISSIPPI VALLEY DENTAL SOCIETY, MARCH 5th, 1873.

The following subject was taken up and discussed :

"*Filling Teeth*—What may the patient reasonably expect shall be accomplished by the operation ?"

Dr. J. Taft, in introduciug the subject, said that those who have the services of the dentist should receive benefit—that which should give relief and comfort. In many cases, however, more is expected than can be realized. The dentist encounters in the practice of his profession many difficulties—difficulties that are not apparent to the uninitiated. These difficulties modifiy the character of operations, and influence more or less the result attainable in them.

The patient and operator are alike subject to unfavorable conditions, which vary exceedingly. That which under one class of circumstances and conditions can be well, and easily done, will, under others, be an entire failure, or only be accomplished with great difficulty and in an imperfect manner.

Some teeth are so defective in structure, that the most that can be done for them by any operation will only retard decay. It was impossible to prevent decay in some teeth even by good filling ; there were local and systemic influences which operated injuriously to the teeth. These things should be taken into consideration, and it was only that patient who understood the modifying circumstances, who could form a just estimate of what can reasonably be expected. It was the dentist's duty to so educate, and give him correct views as to what he should expect.

Dr. McKellops thought patients expected a good deal more than they get.

Dr. Knapp stated that many operations which he expected to last did not turn out well, but he believed that patients could be reconciled to have second and even third fillings in order to save the natural teeth. He thought if dentists did their best they could safely promise patients the use of their natural teeth through life, subject of course to some modification.

Dr. Frank Hunter said, " If we can not give them the assurance that their teeth can be preserved, the question is not answered.

Dr. Harroun thought patients expected too much, yet a great deal lay with the dentist as to what they should reasonably expect. He thought they could expect to retain their teeth, but in his practice he insisted upon the most scrupulous care, cleanliness, and regular and frequent examinations of the mouth and teeth, to see that his patients were careful.

Dr. Rehwinkel called to the chair Dr. Horton. He believed patients expected too much, which was greatly owing to the dentists themselves. Some promised that fillings would last forever and a day, disregarding thereby all considerations of systemic and local causes operating against a healthy status. He had no doubt there were dentists present who observed causes operating through families, destructive of the teeth, and that it was necessary to provide against them. With such knowledge he could tell his patients what to expect, and raise and lower the standard of expectation. Every good dentist knew when the patient went out with the filling in his tooth that all control over it by the dentist ceased. Physiological and pathological causes operate, which may entirely alter the result reasonably expected, and they could not always be forseen. That was exactly where the trouble came in, and patients should be so instructed and informed.

Dr. Wells thought they sometimes discouraged patients when better results might be expected. When a tooth was in good condition to start with, the patient had a right to expect from a careful filling that it would last from two to five years. He instanced teeth decayed below the gums saved by the improved practice of the present day, to show the length patients could go in expectlng useful results from careful filling, and spoke of his own encouragement from experience of saving badly decayed teeth.

Dr. Corydon Palmer thought that as a rule patients did not expect enough. The wholesale destruction of the natural teeth all over this land was sufficient evidence of this to him.

If taught to expect better things the people would not submit to have their teeth extracted ; would not consent to part with them on any consideration. He thought dentists should be true to themselves in this matter, and tell patients what they might expect.

Dr. James Taylor thought his duty ended when he filled the tooth ; then the patient took all the responsibility for the future care of it. As a physician gives medicine for a specific purpose, he expected a certain result, yet there were conditions occuring which modified the result. All this was different with the dentist.

Dr. Horton was of the opinion that people can expect truth when they have the capacity to understand it. There certainly should be an effort to have people understand, and for that purpose there are two parties concerned, the operator and the patient.

For his part he believed in taking hold, and making the very best work, but there were some dentists who seemed to lack the decision to take hold and perform difficult operations, and therefore they failed. They shirk responsibility.

There were some people who insisted on having amalgam fillings, but he maintained that they should be convinced of their error, and that dentists should absolutely refuse to do inferior work. When under-age people come for treatment, their parents or guardians should be sent for, and matters so explained to them that nothing but good work would be received. It was important too that the operator should keep his temper, for he would have much to try it by the ignorance and want of appreciation on the part of patients.

Discussion closed.

On motion of Dr. H. R. Smith, the Association next took up the subject of "Dental Legislation : Is it practicable ? What benefits to the public and the profession may be expected by enforcing such laws ?"

Dr. H. A. Smith doubted if it were practicable. There was an impression abroad that there was too much legislation—class and special legislation. He thought that public opinion regulated evils. He thought legislation for dentistry impracticable, because ever since the law was framed there had been a constant effort to modify it.

Dr. J. Taft took issue with the gentleman. He thought Dr. Smith yielded too much. It was in no sense class legislation, nor could he see that there was any public opinion regarding it as such. The law is an advantage to the dentist, but it exposed the quack, and stripped him of his pretensions. Since it was passed a rapid progress has been made. Hundreds in the profession had studied up, and made progress in order to meet its requirements.

The idea that it was for the advantage of a class or for a few was all wrong. On the contrary it was for the general good—for the dentist as well as for the people, by putting a check upon the mischief done by bad dentists, and by stimulating higher and better efforts and attainments. It had been charged against the law that it raised fees. Nothing could be more untrue, for it is notorious that the best men do not get the best prices. The law did not effect it one way or another.

Dr. J. Taft maintained that the law could not only be made operative, but that it *is* operative, not in a punitory sense, but in preventing the practice of inefficient dentistry. The law was not perfect by any means, yet such as it was there were some in the profession who opposed it. They were to be pitied, perhaps sympathized with, and yet its requirements were such that any dentist could comply with them. Many have already done so, and some have been rejected by the State Board of Examiners, but have gone back and prepared themselves by study, returned again, and were accepted. Such men will stand well and rise in their profession.

After some remarks by Dr. Osmond, H. A. Smith, E. C. Hamilton, and Horton, relative to the form of examinations, the subject was passed.

---

Wounding the tissues beyond the apicial foramen, carrying the filling beyond this point, or imperfect removal of the pulp are errors to be avoided in the treatment of pulpless teeth.

